# Somatic Stem Cells and Their Dysfunction in Endometriosis

**DOI:** 10.3389/fsurg.2014.00051

**Published:** 2015-01-06

**Authors:** Dusan Djokovic, Carlos Calhaz-Jorge

**Affiliations:** ^1^Instituto de Medicina Molecular, Faculdade de Medicina, Universidade de Lisboa, Lisbon, Portugal; ^2^Serviço de Obstetrícia e Ginecologia, Centro Hospitalar de Lisboa Ocidental, Hospital de São Francisco Xavier, Lisbon, Portugal; ^3^Clínica Universitária de Obstetrícia e Ginecologia, Faculdade de Medicina, Universidade de Lisboa, Lisbon, Portugal; ^4^Departamento de Obstetrícia, Ginecologia e Medicina da Reprodução, Centro Hospitalar de Lisboa Norte, Lisbon, Portugal

**Keywords:** somatic stem cells, endometriosis, pathogenesis, endometriosis markers, drug target

## Abstract

Emerging evidence indicates that somatic stem cells (SSCs) of different types prominently contribute to endometrium-associated disorders such as endometriosis. We reviewed the pertinent studies available on PubMed, published in English language until December 2014 and focused on the involvement of SSCs in the pathogenesis of this common gynecological disease. A concise summary of the data obtained from *in vitro* experiments, animal models, and human tissue analyses provides insights into the SSC dysregulation in endometriotic lesions. In addition, a set of research results is presented supporting that SSC-targeting, in combination with hormonal therapy, may result in improved control of the disease, while a more in-depth characterization of endometriosis SSCs may contribute to the development of early-disease diagnostic tests with increased sensitivity and specificity. *Key message*: Seemingly essential for the establishment and progression of endometriotic lesions, dysregulated SSCs, and associated molecular alterations hold a promise as potential endometriosis markers and therapeutic targets.

## Introduction

Somatic stem cells (SSCs), also known as adult or tissue-specific stem cells, play a key role in the regulation of adult tissue homeostasis and regeneration. They are undifferentiated cells with high-proliferative potential that originate from postembryonic cell lineages and share with the embryonic stem cells two fundamental features: self-renewal capacity and multidifferentiative potential (1). SSCs have been described in different tissues, including the endometria of menstruating and non-menstruating mammalian species (e.g., rodents) (2). In highly regenerative human endometrium, remodeling occurs during each menstrual cycle, after resection, parturition, and in postmenopausal women using hormone replacement therapy (3). Increasing evidence indicates that rare endometrial stem cells (EnSCs), which include epithelial and mesenchymal stem cells (MSCs), are responsible for endometrial remodeling and regeneration [reviewed in Ref. (2, 4, 5)]. Besides, bone marrow-derived stem/progenitor cells are also able to incorporate themselves into the endometrium, contributing to the vascular remodeling (6) or transdifferentiating into endometrial cells (7, 8). In parallel, emerging research results suggest that SSCs of multiple types and their abnormalities substantially contribute to endometrial disorders (5).

We review currently available data regarding the stem cell involvement in endometriosis, giving particular attention to the potential clinical integration of the knowledge stemming from basic science research. Using the key words “stem cell” and “endometriosis,” we performed a literature search in PubMed for the publications written in English language until December 1st, 2014. We detected and analyzed 116 articles, and included 54 publications that strictly fit into the topic (descriptive analyses on human tissues, studies in animal models of endometriosis, *in vitro* assays, and previous reviews). The information from 15 additional articles was incorporated for a more comprehensive contextualization of the data, providing, for instance, the epidemiological data on endometriosis, definitions of the terms used, and more profound explanation of some concepts discussed in previously selected 54 articles (Table 1).

**Table 1:** **Publications included in the review**.

	Focus	Reference	
		Original studies	Reviews
Main data sources (54 publications)	Stem/progenitor cells in (endometrium and) endometriosis	(8–39)	(1–7, 40–54)
Supplementary information sources (15 publications)	Endometriosis		
	Epidemiology		(55)
	Molecular biology		(56–59)
	Angiogenesis		(60, 61)
	Animal model	(62)	
	Biomarkers	(63)	(64)
	Stemness markers		(65, 66)
	Stem/progenitor cells in eutopic endometrium	(67)	(68, 69)

## Stem Cell-Based Theory on the Pathogenesis of Endometriosis

Endometriosis is a gynecological disease histologically characterized by the development and growth of endometrium-like lesions outside the uterine cavity. Hypothetical mechanisms that give origin to the endometriosis lesions include retrograde menstruation, lymphatic and vascular spread, as well as iatrogenic implantation of endometrial cells, celomic metaplasia/induced mesenchymal cell differentiation, and embryonic rests (2). As none of these theories individually explains the etiology of all endometriosis types, combined mechanisms, including still undiscovered tissue-specific pathophysiological processes, have been taken into account. Accumulating research data suggest that EnSCs play critical roles in the remodeling and regeneration of the physiological endometrium (1–5, 7, 9–11, 40–49). In parallel with these observations, stem cells have been also considered to be major players in the pathogenesis of endometriosis (1) and other endometrium-associated diseases (5).

Endometrial stem cells are present in the endometrium *basalis* (12) as well as in the menstrual blood (13). In women affected by endometriosis, significantly increased *basalis* portion is shed in the menstrual flow (11). Complementing the Simpson’s theory of retrograde menstruation, it has been hypothesized that dysregulation and dislocation of EnSCs through the retrograde flux into the peritoneal cavity may result in the proliferation of ectopic endometrium-like tissue (3). Such dislocation of EnSCs may occur, in particular, in the women with obstructive malformations of lower genital tract. Rare neonatal retrograde uterine bleeding that displaces stem/progenitor cells present in neonatal endometrium may be in the root of early-onset endometriosis (50, 51). Human endometrium side-population (SP) cells, which supposedly involve EnSCs, were found capable to generated endometrium-like tissue in immunocompromised NOD-SCID mice upon transplantation beneath the kidney capsule (14). Since EnSCs are expected to differentiate in concordance with their microenvironment (i.e., EnSC niche conditions), the deposition of endometrial fragments containing both EnSCs and the niche cells is likely to be required in naturally occurring endometriosis (1). Indeed, unfractionated human endometrial fragments successfully grow in ectopic sites in many experimental models (46, 62). As an alternative, dislocation of functionally aberrant EnSCs may occur (1). By analogy, traumatic dislocation of EnSCs to the myometrium and altered regulatory mechanisms of the EnSC niche have been taken into consideration regarding the pathogenesis of adenomyosis (3).

Furthermore, abnormal cell migration during organogenesis and differentiation of the female reproductive tract, frequently associated with aberrant expression of *Wnt* and/or *Hox* genes, has been also hypothesized as a possible mechanism of the dislocation of primordial cells (15, 52). After menarche, these cells hypothetically give origin to the endometriosis lesions. Additionally, engraftment of bone marrow-derived MSC was documented in a mouse model of experimental endometriosis (15). Interestingly, in the same experiment, the disease was associated with reduced stem cell recruitment in eutopic endometrium. Thus, not only the EnSCs but also extra-uterine stem cells, transported in the blood or lymph, may contribute to the formation of endometriotic lesions through the process of transdifferentiation into endometrial cells (8, 15).

**Figure 1:**
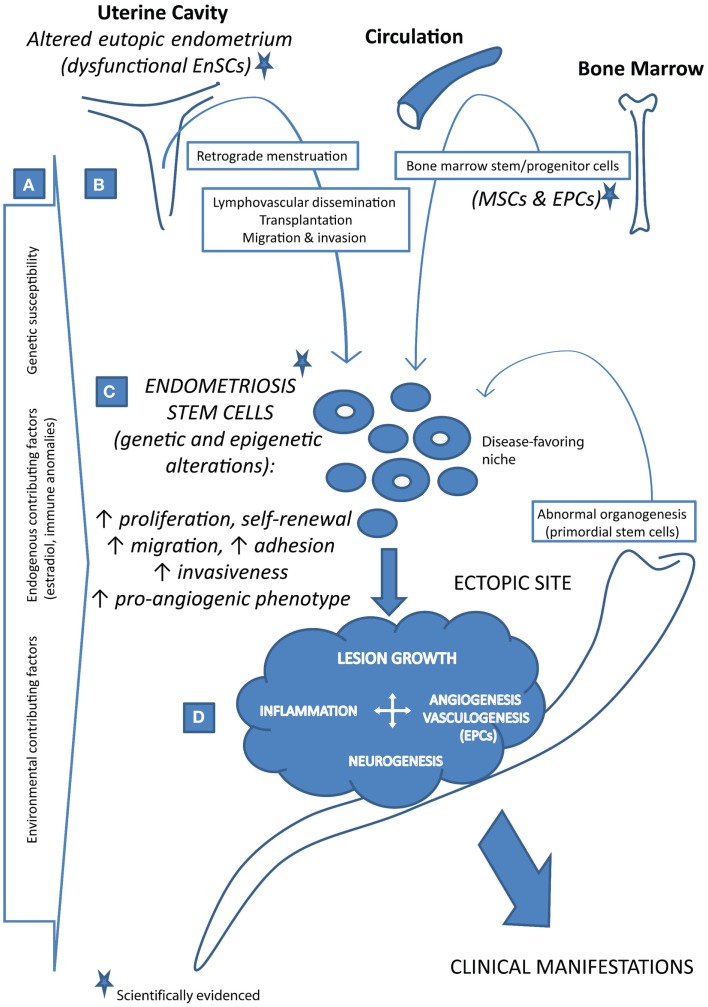
**Stem cell-based concept of the pathogenesis of endometriosis**. **(A)** Endometriosis is a complex, multifactorial disease that develops in persons with genetic susceptibility and in the presence of various endogenous and/or environmental contributing factors. **(B)** Stem cells, which give origin to the endometriosis lesions under disease-favoring microenvironment conditions, may reach the ectopic sites via different routes as such as retrograde menstruation, lymphovascular dissemination, direct transplantation, migration and invasion, and abnormal cell migration during organogenesis. Besides the uterus (i.e., the eutopic endometrium containing endometrial stem cells, *EnSCs*, which are altered in endometriosis patients), bone marrow may also contribute to the endometriosis stem cell pool with its stem/progenitor cells (bone marrow-derived mesenchymal stem cells, *MSCs*, and endothelial progenitor cells, *EPCs*). **(C)** Endometriosis stem cells are dysfunctional due to a range of genetic and epigenetic alterations, displaying increased self-renewal, survival, and aggressive phenotype. **(D)** Upon the establishment, growing lesions require and induce angiogenesis as well as the vasculogenic blood vessel formation from EPCs. Together with inflammation and innervation, vascularized growth of endometriosis lesions results in the appearance of the disease clinical manifestations.

Taken together, different concepts have emerged with reference to the putative stem cell involvement in the pathogenesis of endometriosis. As depicted in Figure 1, both endometrium and extra-EnSC sources were suggested. The stem cell positioning in the ectopic sites was mechanistically attributed to the plausible pathophysiological events such as retrograde menstruation, trauma, lymphovascular dissemination, or aberrant cell migration during reproductive tract organogenesis. In all these scenarios, abnormal stem cell regulation, associated with disease-favoring genetic and epigenetic alterations, constitutes a decisive factor for the disease onset.

## Evidences Supporting Stem/Progenitor Cell Contribution to Endometriosis

Beginning in the late 1990s, several studies appeared indicating a monoclonal origin of ovarian endometrial cysts and individual glands of peritoneal endometriotic lesions, which are, in contrast, polyclonal (16–21). These data did not only suggest a single-cell derivation of ovarian endometriomas but also the possibility that multiple precursor of peritoneal lesions might develop from a single stem/progenitor cell (2). Evidence-based consolidation of the stem cell concept for pathogenesis of endometriosis has been subsequently favored by different descriptive studies on patient tissues and experiments performed *in vitro* or in animal models.

Putative SSCs have been identified in human and animal endometrium through diverse methods focused on the stem cell clonogenicity (i.e., colony formation capacity), label-retaining, “side-population” phenotype, undifferentiation marker expression, or cellular differentiation (1–5, 7, 9–11, 40–49, 68, 69). Briefly, clonogenicity is defined as a single-cell ability to produce a colony consisting of its daughter cells, which are identical to the mother cell. Clonogenic or colony formation assays are cell survival *in vitro* tests that examine the clonogenic ability of every cell in a population. Since the colony-forming ability is a major stem cell feature, observations of colony-forming activity within the epithelial as well as stromal cell populations of endometrium have been indicative of putative stem/progenitor cell presence (3, 48). The concept of SSCs as rarely dividing cells, which retain a DNA synthesis label 5-bromo-2′-deoxyuridine (BrdU), has been also used in endometrium studies. In contrast to the label retention in the immature progenitors, BrdU is diluted in more mature cells due to higher mitotic activity. By identifying and characterizing endometrial label-retaining cells (LRCs), the experiments using this approach did not only point the existence but also the localization of putative SSCs in both endometrial glands and stroma (68, 69). Identification of endometrial “SP” cells has provided additional evidence (9). In flow cytometry, SP cells represent a small cellular sub-population with dye-effluxing properties. These cells, identified in many tissues and found to exhibit some stem cell-like features, efflux DNA-binding dye Hoechst 33342 by the activity of the multi-drug resistance (MDR) genes (e.g., ATP-binding cassette transporter G2, ABCG2). In the case of endometrium, ABCG2+ cells have been demonstrated to reside preferentially in the vessel wall of the endometrial small caliber vessels, holding capacity to differentiate into glandular, stromal, endothelial, as well as smooth muscle cells (9). Different methods used for SSC identification and isolation result in the selection of distinct cell population. Apparently, various SSC types exist. Their phenotypes, functions, and hierarchical relationship remain to be fully elucidated.

However, even before the first identification of the cellular populations with the characteristics of SSCs in endometrium, Osuga et al. documented in 2000, an increase in the concentration of stem cell factor (SCF) in the peritoneal fluid of women with vs. without endometriosis (22). SCF, also known as steel factor, is a ligand of c-KIT receptor (i.e., *c-KIT* proto-oncogene product), which is present on the surface of hematopoietic stem cells and other immature cells (65). SCF binding to c-KIT promotes cell survival, proliferation, and differentiation. In parallel with the cellular differentiation, *c-KIT* expression decreases. This undifferentiation marker was found up-regulated in various cancers (65), as well as in the lesions of endometriosis (23). In addition, some endometriotic lesions over-express T-cell leukemia/lymphoma protein 1A (*TCL1A*) (24), a known marker of immature T- and B-cells and another proto-oncogene causally associated with T-cell leukemia in humans (66). Moreover, endometriosis lesions sometimes over-express Musashi-1, a RNA-binding protein related with the cell fate determination of neural and epithelial progenitor cells (21). Fundamentally important for the self-renewal of embryonic stem cells, undifferentiated embryonic cell transcription factor 1 (UTF1) (24), octamer-binding transcription factor 4 (OCT-4) (23), sex-determining region Y-box 2 (SOX2), and Nanog homeobox (NANOG) (25) are additional undifferentiation markers identified in ectopic endometrioid tissues. The list of stemness-related genes enhanced in endometriosis is continuously expanding with the identification of novel markers, such as importin 13 (IPO13) (26). Collectively, the analyses of undifferentiation markers strongly indicate the existence of immature, undifferentiated progenitors in the endometriotic lesions.

In favor of this hypothesis, aberrant telomerase activity and greater telomere content have been observed in endometriotic lesions (56) and patient peripheral blood (27, 57). These findings may be associated with the immortality of endometriosis stem cells. Besides, Silveira et al. evidenced in a group of eight women affected by endometriosis that chromosomal imbalances were shared by epithelial and stromal cells of endometriosis lesions collected from different anatomical structures of the same patient (28). Oppositely, eutopic endometria of the studied women were characterized by normal karyotypes. In the majority of cases, positive immunostaining for stemness-related markers was detected in isolated epithelial and stromal cells from both eutopic endometria and ectopic endometrium-like tissues. Thus, the study further strengthened the hypothesis that endometriosis lesions have clonal origin with the putative involvement of stem/progenitor cells.

Complementing the knowledge that arose from the analyses of stemness-related markers in endometriosis, Chan et al. performed *in vitro* functional assays and demonstrated that ovarian endometriotic cysts contain a cellular subset that displays SSC features: (1) colony-forming activity, (2) self-renewal *in vitro*, and (3) multipotency (29). The study highlighted the presence of both epithelial and stromal progenitor cells. Colony-forming activity was observed in 0.09% of epithelial and 0.13% of stromal cells. Purified epithelial cells as well as the stromal cells formed large and small colony-forming units (CFUs). Exhibiting substantial self-renewal ability, it was assumed that large epithelial and stromal CFUs had originated from putative SSCs. On the other hand, small CFUs propagated less than the large CFUs. This is consistent with their hypothetical origin from transit amplifying cells which rather differentiate than proliferate. In addition, epithelial small CFUs expressed ovarian epithelial markers while it was only sporadically observed in the large CFUs. Stromal CFUs expressed both fibroblast markers and, importantly, three SSC markers (SALL4-like 4, CD133, and Musashi-1). Finally, the multipotency of stromal large CFUs was proven since they were able to differentiate into four mesenchymal lineages when cultured in appropriate media. Although the identification of stem cells through *in vitro* assays should be critically scrutinized due to the changes in cell biology that may occur under artificial experimental conditions, Chan et al. made with this study a significant step forward in addressing the involvement of stem cells in the formation of endometriotic lesions.

A growing body of evidence also indicates that bone marrow-derived MSC and endothelial progenitor cells (EPCs) contribute to the pathogenesis of endometriosis (10, 30, 31, 53). Cyclic repopulation of endometrium with bone marrow-derived stem cells probably represents a physiologic process, being in part responsible for the stromal regeneration. Although uncommonly, these cells can transdifferentiate into epithelial cells (<0.01%) (7). By analogy, ectopic transdifferentiation might give origin to some endometriotic lesions. Bone marrow-derived MSCs that have previously colonized endometrium may be displaced by retrograde menstruation while direct mobilization of circulating MSCs to the ectopic sides may be driven by a combination of multiple chemoattractants. Thereafter, MSC survival, self-renewal, and lineage-commitment occur depending on the environmental conditions. Hypothetical promoters of ectopic transdifferentiation include endometriosis-associated hormonal and cytokine abnormalities. Serum of women with endometriosis possesses factors that enable MSC transformation into endometrial-like cells and glands (30). *In vitro*, MSCs cultured with sera of women with mild, moderate, and severe endometriosis differentiated into endometrial-like cells and the differentiation rate increased among the three groups from 30 ± 25.8% (mild disease) to 45 ± 29.9% (moderate endometriosis) and 75 ± 37.9% (severe disease form) (30). *In vivo*, in hysterectomized mice, bone marrow MSCs showed the ability to engraft endometrial implants, while in the women transplanted with male bone marrow, donor MSCs, recognizable by the Y chromosome, generated endometrium *de novo* (10). Although ectopic transdifferentiation might sporadically lead to the onset of endometriosis, bone marrow-derived MSCs rather contribute to the growth than initiate the endometriotic lesion. As presented with more details below, the expression of pro-inflammatory cytokines, migration markers, and pro-angiogenic factors is increased in ectopic vs. eutopic MSCs (32, 33). Despite the fact that the grade of MSC contribution to the lesion proliferation requires further clarification, these cells participate in abnormal immune responses and are capable to enhance endometriosis-driven neovascularization. Sakr et al. have demonstrated the shift of MSC engraftment from uterine endometrium to the lesions in a mouse model (15). To promote its development, endometriosis recruits MSCs, competing with eutopic endometrium for the supply of bone marrow-derived cells. In this way, endometriosis additionally interferes with endometrial regeneration, function, and fertility.

Regarding the bone marrow-derived EPCs, up to 37% of the endothelium in the endometriotic lesions originates from these cells (53). Independent of the exact cause(s) and mechanism(s) that lead to the establishment of endometriosis, the growth of its lesions require neovascularization (6, 34, 35). As we and others previously and extensively reviewed (53, 60, 61), endometriosis triggers the angiogenic switch, i.e., the imbalance between up-regulated pro-angiogenic stimuli and suppressed angiogenic inhibitors. Sprouting of new capillary vessels from pre-existing vasculature (angiogenesis) as well as *de novo* generation of blood vessels from undifferentiated progenitors (vasculogenesis) maintain the endometriotic lesions and promote their growth (34–37, 53). In C57BL/6 mice, the disease did not increase the level of EPCs in the blood, bone marrow, and spleen (37). However, vasculogenic establishment of vessel networks from EPCs was documented to represent an integral process in the pathogenesis of endometriosis (37, 53). Endometriotic lesions show increased expression of stromal-derived factor 1 alpha (SDF-1α) and vascular endothelial growth factor (VEGF) (61), which are the main EPC chemoattractant and mitogen, respectively. Hypoxia, inflammation, tissue injury, and estrogen receptor expression promote the EPC mobilization and recruitment (61). EPCs contribute to the vascular network extension, especially in the initial stage of endometriosis establishment. Importantly, pharmacological treatments that suppress EPCs, inhibit the lesion growth in endometriosis-bearing mice (37, 53).

To sum up, ectopic endometrium-like tissues, in both animal models and women affected with endometriosis, contain cellular subpopulations exhibiting stem cell characteristics as indicated by specific gene expression and/or *in vitro* functional assays. Bone marrow stem/progenitor cells are actively present as well. Further characterization (genetic, phenotypic, and functional) of putative endometriosis initiating (stem/progenitor) cells is required while their ability to form endometriosis lesions *in vivo* will be the ultimate proof. Even though the stem cell is a major player, not only the presence of such a cell but also its complex microenvironment (niche) and other contributing factors/pathophysiological events are likely to be essential in the establishment of endometriosis (Figure 1).

## Distinguishing Stem Cell Characteristics in Endometriosis

Although endometriosis-associated stem/progenitor cells remain to be fully characterized, currently available data indicate that they are genetically and phenotypically different in comparison with their physiological analogs. The stem cells from lesions vs. eutopic endometrium possess some distinguishing features while the differences have been also identified between eutopic stem cells from affected vs. disease-free women.

First of all, some stemness-related genes are preferentially expressed in endometriosis lesions (e.g., *UTF1*, *TCL1A*, *ZFP42*, and *SALL4*) and other undifferentiation markers (e.g., *GDF3*) show higher frequency in eutopic endometrium (24). This indirectly suggests that the mechanisms determining the self-renewal rate and stem cell fate are dysregulated in endometriosis. Consequently, altered stem cell behavior favors the disease onset. Known chromosomal aberrations, impaired DNA methylation, histone modifications, and imbalance of microRNA (miRNA) expression that are associated with the phenotypic changes of endometriosis stem cells have been recently reviewed by Forte et al. (40). Particularly, intriguing findings arose from the studies on miRNA imbalances. Naturally occurring miRNAs are single-stranded non-coding small RNA molecules that function as post-transcriptional gene repressors contributing, in this way, to the determination of cell identity and fate (58). Their alterations have been documented in endometriotic lesions by real-time reverse transcription PCR, TaqMan real-time PCR, and microarrays (40). Dysregulated miRNAs apparently influence the endometriosis stem cell phenotype in various manners. For instance, impaired miR-145 function, observed in endometriosis patients, promotes the stemness itself and enhances the stem cell invasiveness (58) while down-regulated miRNA-199a-5p contributes to the endometriosis progression by promoting pro-angiogenic factor production by endometrial MSCs (34).

Despite the complexity of known and unknown molecular phenomena underlying the phenotypic changes exhibited by endometriosis stem cells, these changes involve five main functional alterations: increases proliferation, migration, adhesion/invasiveness, pro-angiogenic factor production, and dysregulated expression of certain immune modulators (13, 31, 33, 36, 38, 59). In two independent studies, Kao et al. (31) and Moggio et al. (36) evidenced a higher proliferative, migratory, and pro-angiogenic potential of ectopic MSCs in comparison with the eutopic MSCs from the same patient or control MSCs from women without endometriosis. Kao et al. (33) equally witnessed the ectopic MSC to present augmented invasiveness. These findings coincided with significantly higher interleukin-1 beta (IL-1β) and ciclo-oxigenase-2 (COX-2) levels in ectopic MSCs compared with eutopic MSCs. Additionally, treatment with a COX-2 inhibitor induced apoptosis and suppressed migration and invasiveness of adenomyosis MSCs, but not of eutopic MSCs (38). Thus, eutopic and ectopic MSCs are substantially different in terms of functionality. A recent study compared eutopic and ectopic MSCs by analyzing their phenotypes and gene expression of pattern recognition receptors (PRRs), pro- and anti-inflammatory cytokines, migration markers, and angiogenesis factors (32). The findings indicated increased levels of PRRs such as toll-like receptors and collectins, down-regulated anti-inflammatory TGFβ and increased transcription of pro-inflammatory cytokines (IL-6, IFNγ), migration markers (MMP-2, -3, -9), and pro-angiogenic VEGF in ectopic vs. eutopic MSCs. These results suggest that pathogenic behavior of ectopic MSCs may be partially responsible for attenuated immunosuppression and enhanced angiogenesis in endometriosis.

Furthermore, menstrual blood-derived stromal stem cells from endometriosis patients vs. healthy women possess altered morphological features, higher CD9, CD10, and CD29 expression, increased proliferation potential and invasiveness, and distinct ability to express certain immune modulators when co-cultured with allogeneic peripheral blood mononuclear cells (13). If we accept that retrograde menstruation is a major mechanism that disseminates endometrial cells to the peritoneal cavity, these data may explain why endometriosis does not affect all women with retrograde menstrual flux, but only some of them (those with altered stem cell function). More broadly, impaired stem cell function is detrimental, regardless the mechanism used by endometrium- or bone marrow-derived stem cells to engraft the ectopic sites. Increased survival, resistance, and phenotypic shift to a more aggressive behavior are absolutely essential as far as currently available evidences imply.

Finally, highly variable extension and clinical expression of endometriosis could be related with the type of dysfunctional stem cell responsible for its establishment. There are distinct differences between EnSCs and bone marrow-driven MSCs. Both EnSCs and MSCs produce immune-modulatory effects, but they show significant differences in many immune/inflammatory pathways at the protein and transcriptome levels (67). A number of other, stemness- and cancer-related genes are differentially expressed between these cells. Regarding the pro-angiogenic potential, MSCs express higher levels of VEGF while EnSCs express substantially higher levels of platelet derived growth factor (PDGF) and angiopoietin-1 (Ang1) (67). This implies that EnSCs are more angiogenic than MSCs since they are capable to trigger alternative angiogenic pathways. Specific stem cell type or even subtype that initiates and/or predominantly mediates endometriosis development may be indeed decisive with respect to the evolution and clinical manifestations of the disease. The invasiveness of endometriosis hypothetically increases with the increase of the initiating cell aggressiveness, immune-modulatory, and pro-angiogenic abilities.

## Clinical Perspectives

Endometriosis affects approximately 8% of women of the reproductive age (55), represents a major cause of chronic pelvic pain and female infertility, and tremendously burdens the health systems around the world. Currently, two major problems hinder the medical assistance to the women suffering from endometriosis: the lack of appropriate biomarkers useful in early diagnosis and the inexistence of conservative (medical) treatments with long-lasting effect. Further characterization of the stem cell populations in endometriosis is challenging and may open up possibilities for rational development of novel and improved diagnostic and therapeutic strategies.

Non-invasive tests are greatly needed for the early detection of endometriosis in women suffering of chronic pain and/or infertility with normal ultrasound findings. A test based on the use of 28 potential markers has been recently proven as a highly sensitive and specific tool to establish the diagnosis of ultrasound-undetectable endometriosis (63). Complementing this encouraging, but complex multi-marker approach, it will be interesting to assess whether the blood levels of circulating stem/progenitor cells are specifically different in patients to serve as indicators of endometriosis and/or treatment efficacy. Increased blood levels of EPCs or MSCs have been evidenced in some animal models (53), but still there is no consistent overall information (35). The tests of peripheral blood miRNA levels may be also useful. As previously stated, important miRNA alterations have been documented in endometriosis, including those associated with stem cell dysfunction. In contrast to the serum proteins, circulating miRNAs, as nucleic acids, can be amplified. Consequently, their alterations may be detected with better sensitivity and specificity (64).

Regarding the therapeutic usefulness, targeting stem cells may be beneficial in accordance with their fundamental role in endometriosis as well as with few seminal, but significant experimental results. Potential usefulness of the SSCs from eutopic endometrium has been already demonstrated in the treatment of several chronic diseases (e.g., diabetes and Parkinson’s disease) (54). In contrast to these clinical conditions wherein EnSCs may be engaged to provide specific differentiated cells and tissue regeneration, suppression of stem cell recruitment and activity is required in endometriosis. In a mouse model of endometriosis, a selective estrogen receptor modulator bazedoxifene, administered with conjugated estrogens, dramatically reduced the recruitment of bone marrow-derived MSCs to the lesions, promoted MSC engraftment in eutopic endometrium and resulted in regression of endometriosis (15). Some constituents of tobacco smoke also impair the stem cell influx to endometriosis lesions (39); however, their pharmacological formulation is not yet available. Moreover, sorafenib, a tyrosine kinase inhibitor and potent VEGF signaling suppressor approved for the treatment of different cancer types (60), was found able to inhibit endometriosis MSC proliferation, migration, and pro-angiogenic phenotype (36), while AMD3100, an antagonist of SDF-1α/CXCR4 signaling, decreased both EPC number and vascular density in murine intraperitoneal endometriotic lesions (37). Although few in number, all of these concrete examples imply that stem cell targeting, in combination with hormonal therapies, may contribute to more effective medical treatment and prevention of relapses in endometriosis-affected women. The strategies that will certainly deserve consideration in future research include targeting of altered molecular mechanisms in and outside the endometriosis stem cells, which promote their recruitment, adhesion, survival, self-renewal, and pro-angiogenic behavior.

## Conclusion

The involvement of different kinds of SSCs in pathogenesis of endometriosis and their dysfunction have become increasingly evident. With the stem cell-based concept, it is possible to integrate most of previously proposed theories regarding the pathogenesis of endometriosis in a single and meaningful system. A more in-depth comprehension of distinguishing stem cell characteristics and altered regulation in this prevalent disease is needed. Such research could contribute to the development of new and improved diagnostic and therapeutic modalities for patients suffering from endometriosis.

## Conflict of Interest Statement

The authors declare that the research was conducted in the absence of any commercial or financial relationships that could be construed as a potential conflict of interest.

## Abbreviations

ABCG2, ATP-binding cassette transporter G2; Ang1, angiopoietin-1; BM-MSC, bone marrow-derived mesenchymal stem cell; BrdU, 5-bromo-2′-deoxyuridine; CD, cluster of differentiation; CFUs, colony-forming units; COX-2, ciclo-oxigenase-2; CXCR4, C–X–C chemokine receptor type 4; EnSC(s), endometrial stem cell(s); EPC, endothelial progenitor cells; GDF3, growth differentiation factor-3; IFNγ, interferon gamma; IL-1β, interleukin-1 beta; IL-6, interleukin-6; IPO13, importin 13; LRCs, label-retaining cells; MDR, multi-drug resistance genes; miRNA, microRNA; MMP-2, -3, -9, matrix metalloproteinases 2, 3, 9; MSC, mesenchymal stem cell; NANOG, Nanog homeobox; OCT-4, octamer-binding transcription factor 4; PCR, polymerase chain reaction; PDGF, platelet derived growth factor; PRRs, pattern recognition receptors; SALL4, sal-like 4; SCF, stem cell factor; SDF-1α, stromal cell-derived factor 1 alpha; SOX2, sex-determining region Y-box 2; SP, side-population cells; SSC(s), somatic stem cell(s); TCL1A, T-cell leukemia/lymphoma protein 1A; UTF1, undifferentiated embryonic cell transcription factor 1; VEGF, vascular endothelial growth factor; ZFP42, zinc finger protein 42 homolog.
